# Why the Indian Subcontinent Holds the Key to Global Tiger Recovery

**DOI:** 10.1371/journal.pgen.1000585

**Published:** 2009-08-14

**Authors:** Samrat Mondol, K. Ullas Karanth, Uma Ramakrishnan

**Affiliations:** 1 National Centre for Biological Sciences, Tata Institute of Fundamental Research, Bangalore, India; 2 Wildlife Conservation Society, Bronx, New York, United States of America; 3 Centre for Wildlife Studies, Bangalore, India; Stanford University, United States of America

## Abstract

With only ∼3,000 wild individuals surviving restricted to just 7% of their historical range, tigers are now a globally threatened species. Therefore, conservation efforts must prioritize regions that harbor more tigers, as well try to capture most of the remaining genetic variation and habitat diversity. Only such prioritization based on demographic, genetic, and ecological considerations can ensure species recovery and retention of evolutionary flexibility in the face of ongoing global changes. Although scientific understanding of ecological and demographic aspects of extant wild tiger populations has improved recently, little is known about their genetic composition and variability. We sampled 73 individual tigers from 28 reserves spread across a diversity of habitats in the Indian subcontinent to obtain 1,263 bp of mitochondrial DNA and 10 microsatellite loci. Our analyses reveals that Indian tigers retain more than half of the extant genetic diversity in the species. Coalescent simulations attribute this high genetic diversity to a historically large population size of about 58,200 tigers for peninsular India south of the Gangetic plains. Furthermore, our analyses indicate a precipitous, possibly human-induced population crash ∼200 years ago in India, which is in concordance with historical records. Our results suggest that only 1.7% (with an upper limit of 13% and a lower limit of 0.2%) of tiger numbers in historical times remain now. In the global conservation context our results suggest that, based on genetic, demographic, and ecological considerations, the Indian subcontinent holds the key to global survival and recovery of wild tigers.

## Author Summary

Tiger range and numbers have collapsed globally despite substantial conservation efforts. Genetic data quantifying variation from 73 wild tigers in 28 reserves in the Indian subcontinent suggests historically high numbers for tigers, and simulations reveal a signature of a 200-year-old, possibly human-induced decline. Simulations suggest that only 1.7% of historical tiger numbers now persist in peninsular Indian. Our data also reveal that tigers of the Indian subcontinent retain most of the species' genetic diversity, besides this region harbouring maximum diversity of tiger habitats. Overall, the Indian subcontinent appears to be a global hotspot holding the key to any future recovery of wild tigers from both an ecological and genetic perspective.

## Introduction

As top predators, large carnivores strongly shape ecological interactions in biological communities, thus playing a critical role in maintaining their structure and diversity [1],[2]. However, during historical times, increased anthropogenic impacts have driven range collapses and population declines in many large carnivores, thereby engendering significant efforts at species recovery. These efforts have typically aimed at increasing local population sizes and enhancing connectivity between populations using available demographic, ecological and genetic information for the species [3]–[5]. Integration of such datasets is critical for prioritizing conservation efforts.

The tiger (*Panthera tigris*) typifies large carnivores severely threatened by historical anthropogenic impacts. Wild tigers historically occurred across 70 degrees of latitude and 100 degrees of longitude, spanning 30 present-day nations ranging from Armenia to Indonesia, the Russian Far East to the Southern tip of India [6],[7]. This range encompassed a variety of habitats, including taiga and boreal forests, tropical evergreen, moist and dry deciduous forests, alluvial grasslands and mangroves. Historical times have seen a dramatic range collapse of 93% for wild tigers due to habitat loss, prey depletion and direct hunting [7]. Current global estimates of wild tiger populations range from 3000–3500 individuals [7],[8]. The Indian subcontinent is estimated to harbor about 2000 tigers [9], or about 60% of the global wild population, although it retains only an estimated 8–25% of remaining global habitat [7],[9],[10]. These data emphasize the importance of Indian tigers for future species recovery from a demographic perspective. Ecology and population dynamics of tigers in the Indian subcontinent has been reasonably well studied [11],[12], as has been their spatial distribution and habitat diversity [9],[10]. However, the genetic make up and diversity of Indian wild tiger populations have not been examined in a global context.

Limited phylogeographic studies [13],[14] reveal only moderate levels of variation within Indian tigers, in spite of more than half of the global population and the most varied habitat conditions occurring in this region. In order to adequately assess genetic variability of extant Indian tigers, it is critical to obtain as many genetic samples as possible from the varied, disjunct and fragmented tiger habitats. However, because wild tigers are endangered, elusive and difficult to capture, it is difficult to invasively collect sufficient samples such as blood or tissue. We overcome this problem by using genetic samples non-invasively collected from tiger scats to assess genetic variation, phylogeography and demographic history of tigers in the Indian subcontinent.

In this paper we investigate (1) the proportion of global tiger genetic variation harboured by tigers in the Indian subcontinent and (2) the demographic history of tigers in the Indian subcontinent, with a synthesis based on historical population sizes and recent human impacts. We address these questions using 1.26 kb of mitochondrial DNA and 10 microsatellite loci surveyed in 73 individual tigers from 28 different populations, and compare our results to published data from 68 tigers outside the Indian subcontinent.

## Results

### Genetic variation

**Figure 1:**
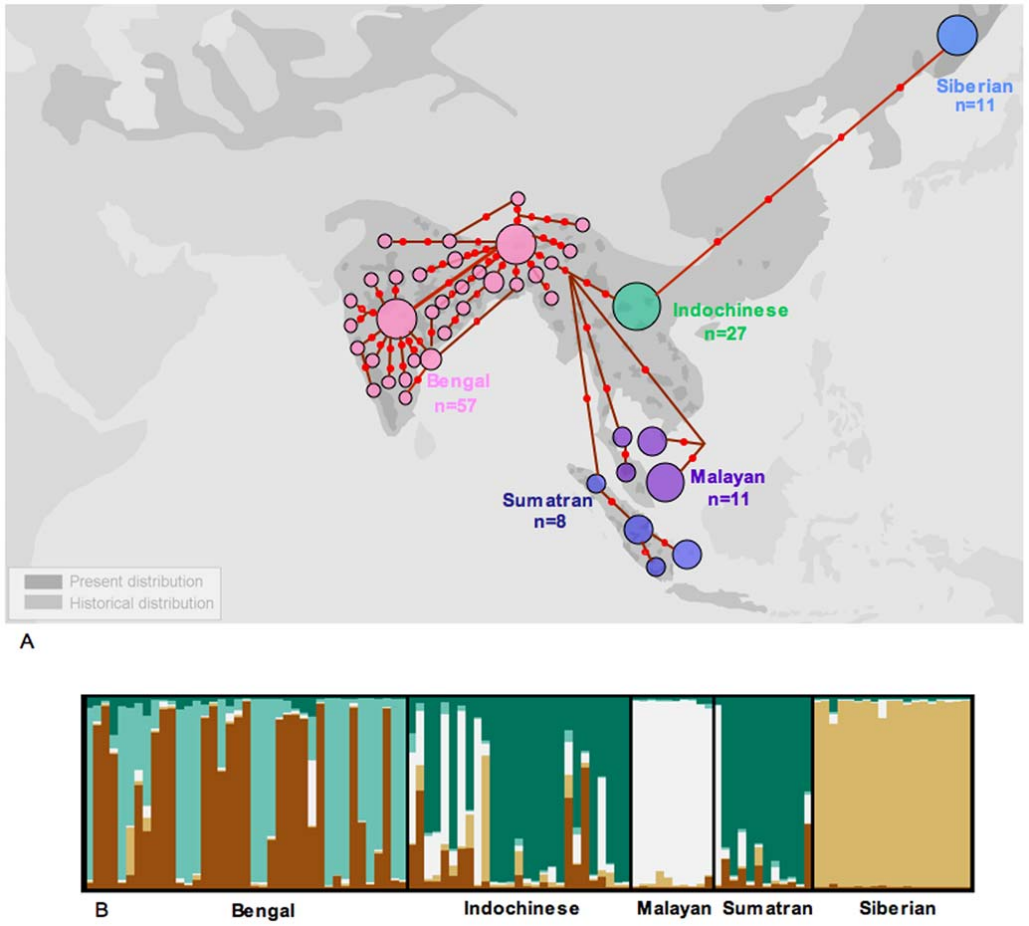
Comparison of genetic variation among all tiger subspecies. (A) A haplotype network based on 1,263 bp of mitochondrial DNA reveals that 76% of global tiger genetic variation (32 haplotypes out of 42) is retained within the Indian subcontinent. Locations of each haplotype within India correspond only approximately to the site of sample collection. 71 samples are included from Myanmar, Nepal, and India. All samples from outside India are from Luo *et al.*, 2004 [13]. (B) The partitioning of microsatellite genetic variation based on 5 common microsatellite loci between different sub-species of tigers, with program STRUCTURE (k = 5). Individuals within the Indian subcontinent are the most variable, indicated by the fact that all 5 colours are present, compared to other subspecies, which retain only one colour (e.g. Siberian tigers). All samples from outside India are from Luo *et al.*, 2004 [13].

Our sampling strategy concentrated on tiger populations living in varied habitats throughout the Indian subcontinent. Using 71 (Table S1) non-invasively collected fecal samples and two tissue samples from across the Indian subcontinent, we investigated genetic variability of Indian tigers using mtDNA and microsatellite loci. Our results reveal that Indian tigers have much higher genetic variation than wild tigers elsewhere. For mitochondrial DNA, 76% of all tiger genetic variability (32 out of 42 haplotypes) is found within the Indian subcontinent (Figure 1A). These results are robust to differences in sample size (67 within India versus 57 outside India). Re-sampling simulations reveal that if only 57 Indian tigers were sampled, we would expect between 18 and 25 haplotypes, much higher than the observed 10 haplotypes. Similarly, five microsatellite loci reveal higher average number of alleles (Table 1), allelic size range (Table 1) and heterozygosity (Table 1) in Indian subcontinent tigers compared to tigers from the rest of the world. Additionally, the program STRUCTURE [15] (based on five loci) illustrates that Indian tigers retain high allelic richness and varied ancestry (Figure 1B). Thus both markers and several analyses suggest higher genetic variability within the Indian subcontinent compared to any other subspecies, as well as all compared to all tiger subspecies outside the Indian subcontinent (Figure 1, Table 1).

**Table 1:** Comparing genetic variation at five nuclear microsatellites.

Subspecies	Observed heterozygosity (S.D.)	Number of alleles (S.D.)	Allelic size range (S.D.)
Bengal (P. tigris tigris)	0.70 (0.16)	12.4 (3.6)	32 (7.7)
All other subspecies (Indo-Chinese, Malayan, Sumatran and Siberian)	0.53 (0.07)	7.2 (1.6)	16 (6.1)
All South-East Asian subspecies (Indo-Chinese, Malayan and Sumatran)	0.56 (0.14)	7.2 (1.6)	16 (6.1)
Indo-Chinese (P. tigris corbetti)	0.57 (0.27)	6.2 (1.5)	14.8 (4.8)
Malayan (P. tigris jacksoni) and Sumatran (P. tigris sumatrae)	0.55 (0.05)	5.8 (1.5)	13.2 (6.1)

Viewed alone, this higher genetic variability observed within the Indian subcontinent is concordant with India being the geographic source for tigers. However, fossil evidence suggests that the tiger evolved in southern China [16]. Phylogenetic and phylogeographic data [13] suggest the genetic antiquity of the Indochinese tigers (very limited samples from South China tigers), and that Indian tigers are of relatively recent origin. Coalescent-based two population models (LAMARC [17]; Indian subcontinent tigers versus Indochinese tigers) based on both mitochondrial DNA and microsatellite data (based on five loci) estimate significantly higher immigration into the Indian subcontinent [MLE_mtDNA_ (maximum likelihood estimate) = 185.8 (44.72, 486.63); MLE_microsats_ = 36.82 (31.17, 40.40)] compared to emigration [MLE_mtDNA_ = 0.19 (0.000001, 59.84); MLE_microsats_ = 13.77 (11.31, 15.32)] out of the subcontinent. Although not conclusive, these independent lines of evidence suggest that Indian tigers are not ancestral to the species.

The higher genetic diversity of Indian tigers could be explained by higher effective population size, due to (1) high levels of population differentiation between tiger populations within the subcontinent due to habitat variability and past fragmentation and/or (2) high historic abundance of tigers in the Indian subcontinent.

We investigated the impacts of population differentiation on our results. We divided our samples into those roughly from the North, Central and South of the Indian subcontinent. Our results reveal a strong signature of population structure for mitochondrial DNA (Table 2), especially between the North and the Central and Southern region. On the other hand, pairwise Fst values (Table 2) for microsatellite data are low. Although the structure plot (Figure 1B) reveals varied ancestry, there is no clear partition of ancestry between regions.

**Table 2:** Genetic differentiation (pairwise F_st_) at mitochondrial genes (1263 bp, below diagonal) and nuclear microsatellites (ten loci, above diagonal, in italics).

	North (n = 10)^2^	Central (n = 11)^2^	South (n = 18)^2^
North (n = 24)^1^		*0.027 (p = 0.063)*	0.041* (p = 0.000)
Central (n = 18)^1^	0.236* (p = 0.000)		*0.019 (p = 0.054)*
South (n = 26)^1^	0.298* (p = 0.000)	0.026 (p = 0.279)	

1 Mitochondrial DNA sample sizes.

2 Microsatellite sample sizes.

We estimated historical effective population size for Indian tigers. Our mitochondrial DNA data suggest population expansion within the Indian subcontinent (Fu's F = −26.33 (p = 0.000); LAMARC: MLE of g = 2859.7 (2092.67, 5549.68), indicating growth). In contrast, the microsatellite data from the same populations indicate population decline (M ratio 0.35 (s.d. 0.08), BOTTLENECK [18]: 7 to all of the10 loci with heterozygote excess depending on the mutational model, LAMARC (based on 10 loci): MLE of g = −20.87 (−24.29, −17.09), indicating population decline). Tests for selective neutrality of the tiger mitochondrial genome revealed evidence for negative selection on the cytochrome b gene (dN/dS within species = 1.9, between species = 0.09, p = 0.00002). Because of negative selection, mitochondrial genetic variation may result in underestimates of historical population size.

**Figure 2:**
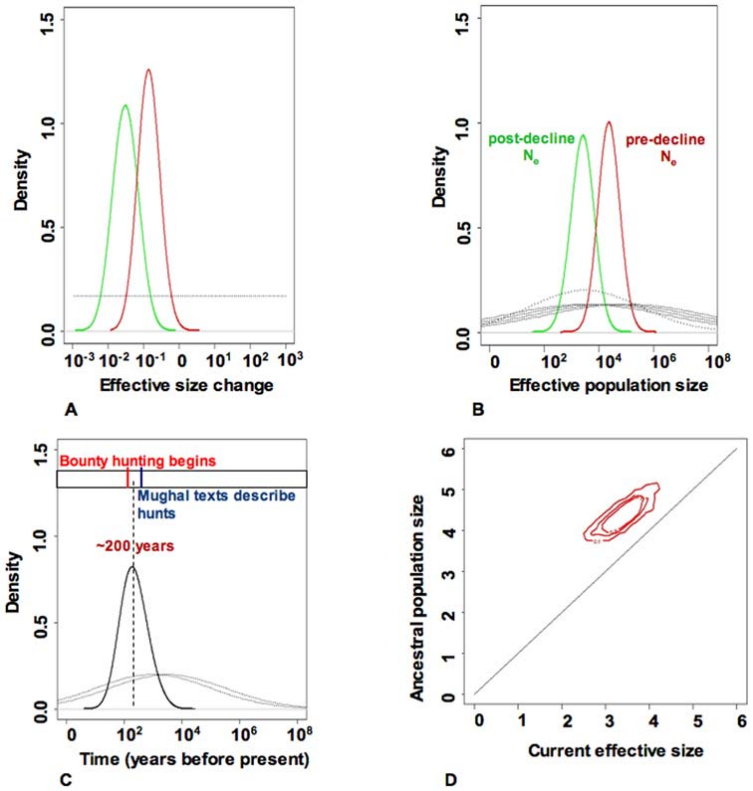
Demographic history of peninsular Indian tigers (*Panthera tigris tigris*). (A) Posterior distributions for population size change based on coalescent simulations for peninsular Indian tigers based on 10 microsatellite loci and the Beaumont method. Red and green curves correspond to the posterior distributions under models of exponential and linear population size change, respectively. The prior distribution is represented by the flat dotted line. Irrespective of various models, there is no support for population increase. For peninsular Indian tigers, the results reveal about 10-fold decrease. (B) Posterior distributions for population size change based on coalescent simulations for peninsular Indian tigers based on 10 microsatellite loci and the Storz and Beaumont method. The posterior distributions for ancestral (red curve) and present (green) effective population size are represented here. The priors are represented by the dotted line (present population) and dashed line (ancestral population). Results confirm that post-decline population size is much smaller than the pre-decline population size. (C) The posterior distribution for the time since the population decline started for Indian tigers (black curve). The priors are shown by the dashed lines. The distribution has a median value at around 200 years. The vertical red and blue lines represent the approximate time since bounty-killing by the British and first written history of tiger hunting by Mughals respectively. (D) The joint posterior distribution of ancestral and present effective population size based on Indian tiger data. The 90%, 50%, and 10% highest probability density (HPD) limits are plotted for the joint distribution of ancestral and current population size on a logarithmic scale. The diagonal line corresponds to stable population size.

Given that tigers from Central and Southern India do not reveal strong subdivision (low and non-significant pairwise F_st_'s for both mitochondrial and microsatellite DNA), we investigate the demographic history of tigers in this region that we refer to as peninsular India (including the states of Madhya Pradesh, Chattisgarh, Maharastra, Karnataka, Tamil Nadu, Andhra Pradesh and Kerala) using coalescent simulations [19],[20]. Models including linear and exponential decline (Table S5 and Table S6, Figure S4 and Figure S5) revealed that the current effective size is about one tenth of the historical effective size, indicating that the Indian subcontinent has lost about 90% of its tigers (Figure 2). As these models do not allow us to investigate the absolute historical effective size as well as the timing of decline, we also used the Storz and Beaumont method to explore recent population decline. Results reveal a strong signal of population decline (91.4% decline, Figure 2), which potentially occurred around 200 years before present.

### Sensitivity analyses

We investigated the sensitivity of our results on demographic history to the number of genetic loci. Coalescent simulations that included 5 wild caught tigers (data from 13) genotyped at 30 microsatellite loci also revealed a very similar extent and timing of demographic decline (Figure S3). Further, using genetic data from tigers across the Indian subcontinent resulted in similar extent of population decline (Figure S2). Our sensitivity analyses re-iterate that the extent of demographic decline and its timing are robust to population structure as well as increased genetic data.

## Discussion

### Genetic variation

An assessment of genetic variation for tigers reveals tigers in the Indian subcontinent retain more than 60% of the genetic variability of the species. In this study, we have taken extreme care to sample Indian tigers in a spatially exhaustive way. However, our results are also conditional on adequate sampling of tiger variation outside the India. Future studies might also include additional sampling of wild tigers outside the Indian subcontinent. Additionally, data from captive tigers of known origin could also be used to investigate discrepancies in genetic variation.

Indian subcontinent tigers could retain higher genetic variation not only because they had high historical population size but also because other tiger subspecies declined more severely in the recent past. Single population models in LAMARC for Indochinese tigers using the microsatellite data also exhibit a signature of recent population decline (MLE of g = −0.881825 (−1.29, −0.560142)), although lower than the estimated decline for Indian tigers. We also quantified the timing and extent of demographic decline, and simulations revealed a relatively recent decline (158 years ago, Figure S6, Figure S7, and Figure S8) of about the same magnitude (90%) as for Central and South Indian tigers. However, the median ancestral effective size for Indochinese tigers was much lower than that of the Indian tigers in central and south India (23,280). The higher ancestral effective size explains the higher genetic variation among extant Indian tigers inspite of recent, human induced decline.

### Demographic history

Data from mitochondrial DNA potentially reveal a signal of demographic expansion, while microsatellite data reveal a signal of a recent population decline. The strong population differentiation for mitochondrial DNA (Table 2) could also result in the observed, expansion-like pattern [21] and the resultant high mitochondrial genetic diversity in this part of the species' range.

Our genetic data in combination with a series of simulation models suggests that prior to historical human impacts, the genetic effective population size for tigers from peninsular India was between 2,964 and 151,008, with a median value of 23,280. Converting this effective population size into a population size suggests that between 7,412 and 377,520, with a median of 58,202 (using N_e_/N = 0.4 [22], where N_e_ is the effective population size and N is the census size) adult wild tigers inhabited peninsular India prior to these human impacts. Given the recent census estimate of around 1,000 adult tigers in peninsular India [9], this corresponds to a median decline of approximately 98% over the last 200 years. Major demographic declines are evidenced by historical hunting records (based on bounty killings) during the Colonial rule, which suggest that over 80,000 tigers were hunted for bounties between 1875 and 1925 [23] across the Indian subcontinent. These data potentially indicate an even higher historical population size for tigers than do our results. However, the demographic decline we detect is in the face of high potential annual growth rates [12], suggesting that hunted individuals far exceeded 57,000 (98% of the ancestral effective size), even in peninsular India. Accounting for population growth rates suggests that our genetic results might be in concordance with historical hunting records. Our estimates of the decline are linked to the assumed N_e_/N ratio of 0.4 [22]. Empirical estimates for mammals suggest a median N_e_/N of 0.6 [24] while theoretical estimates suggest N_e_/N of 0.5 [25]. These estimates would reduce the decline to 97.4% and 97.8% respectively. Alternatively, N_e_/N values lower than 0.4 would accentuate the decline scenario we propose.

It is known that in the last ∼600 years, two major historical events [23] affected tiger populations across the Indian subcontinent: the intrusion and political control of India by Mughal' warriors who were known for their advanced hunting technologies, and subsequently, the establishment of the British Empire, which promoted widespread use of fire-arms, modern technologies and encouraged mass hunting of tigers for bounty and sport. Our results estimate the median timing of decline to 200 years ago, overlapping with the extensive bounty killings, which started around 130 years ago under colonial rule [23]. Historical records indicate that tigers were systematically hunted from Mughal times (around 500 years ago, [23]) and the subsequent colonial rule additionally encouraged bounty-hunting (initially in eastern India) from about 1777 (231 years ago) [23]. It is possible that tiger population declines started during the Mughal empire and accelerating over the last 150 years during colonial rule. The analytical approaches we use in this paper explore relatively simple scenarios of decline. However, additional historical genetic data from trophy or bounty hunted tiger skins might allow investigate as to whether rates of decline in tiger populations have increased relatively recently. Finally, our estimates of the timing of decline are based on an assumed 5-year generation time for tigers [22]. A higher generation time (say 6 years) would result in a younger estimated timing of decline, while a lower generation time would result in an older estimated timing of decline.

### Population structure

The differences between the observed patterns of population differentiation between mitochondrial and nuclear markers could be because of the lower effective size for mitochondrial DNA [26],[27] or due to interactions between mode of inheritance and sex-biased dispersal. Female tigers have smaller home ranges than males, with daughters inheriting the home range of their mothers [28]. This would result in strong population subdivision for maternally inherited mitochondrial DNA. On the other hand, biparentally inherited nuclear (microsatellite) data are consistent with expectations for a large carnivore species that exhibits long-range dispersal movements [28]. Discordant patterns for population differentiation between mitochondrial and nuclear DNA are common for mammals with female philopatry and male-biased dispersal [29]. It is interesting that population subdivision between Southern and Central India is not significant for both mitochondrial and nuclear DNA, suggesting dispersal through a wide range of habitat types as well as relatively recent fragmentation of the formerly contiguous tiger habitats in peninsular India.

### Implications for conservation

Our results are important for global tiger conservation because they suggest that Indian tiger populations are critical for species recovery. However, because tiger habitats in India are often small, disjunct and fragmented, conservation options are limited. Ecological studies [7],[10],[12] have identified a few protected landscapes in India with high tiger densities and potential connectivity. Conservation efforts must prioritize these tiger populations in larger landscapes like the Western Ghats, Central India and the alluvial flood plains in the Himalayan foot hills that support high potential tiger densities, and relatively larger populations.

Genetic diversity retains the history of a species [30],[31], and is vital for survival and future adaptation to changes [32]. Although it is possible that other tiger populations outside of India harboured increased genetic variation in the past, our results show that currently Indian tiger retain the majority of the species' genetic variation. This suggests subspecies-based conservation criteria are inappropriate. Despite having experienced recent demographic declines, extensive habitat loss, extant Indian wild tigers retain 76% of the mitochondrial diversity and 63% of the species' nuclear genetic diversity and are adapted to a greater diversity of habitats [7],[12]. They are thus critically important from demographic, evolutionary and ecological perspectives for future survival and recovery of the species. More than a billion people, afflicted by poverty and yet experiencing rapid economic growth live in India. That Indian tigers have managed to retain their genetic diversity in the face of such high anthropogenic pressure provides some hope for species survival in the future.

## Materials and Methods

### Sampling and DNA extraction

Samples were opportunistically collected from wild individuals living inside protected areas and national parks spanning all over India. We collected 71 fecal samples and two tissue samples from most of the tiger habitat in India. Tissue samples were collected from poached animals with permissions. All samples were collected in sterile vials and preserved in absolute alcohol until processed. To avoid the effects of inbreeding in our analyses, samples were collected spatially far apart within a protected area (at least 15 km apart). DNA extraction and species identification was performed by methods explained in Mukherjee *et al.* [33]. Sampling information is provided in supplementary material (Table S1). We generated mitochondrial DNA data for 54 samples and STR data for 58 samples. In addition to the samples collected in this study, we used genetic data from all tiger subspecies (Indo-Chinese, Malayan, Sumatran and Siberian, in addition to Indian tigers) from Luo *et al.* [13]. All genetic data for tigers outside the Indian subcontinent (as presented in Figure 1) are from Luo *et al.* [13]. We only considered data from tigers of known wild origin. A total of 57 tiger mitochondrial DNA and 68 STR data have been used for comparison in this study.

### Marker selection and amplification

We designed tiger-specific mitochondrial DNA primers. Polymorphic regions were ascertained using tiger mitochondrial sequences across all subspecies based on Luo *et al.* [13] (Figure S1). These primers were then standardized for fecal DNA samples. A total of nine primer sets were designed, and used to amplify1263 bp from Indian tiger fecal samples. Primers sequences are presented in supplementary information (Table S2).

We selected ten felid-specific microsatellite loci based on PCR success rate, amplicon size, number of alleles and the level of observed heterozygosity (H_obs_) in Indian tigers (Table S3). All samples were genotyped at this panel of nine dinucleotide and one tetranucleotide microsatellite locus described initially in the domestic cat [34].

The mitochondrial regions were amplified in 10 µl volume reactions, cleaned by Exo-Sap mixture (NEB) and sequenced from both ends on an ABI 3100XL capillary sequencer. To monitor possible contamination, PCR blanks were included in all experiments.

Amplification for all the microsatellite loci was done using a multiplex approach.

A modified multiple tube approach, combined with a quality index approval was used for data quality management to account for the varying quality and quantity of DNA obtained from non-invasive sources. The complete genotyping process was repeated three times for all samples, and only those loci with quality index ≥0.75 were included in the analysis [35].

### Data analyses

Genetic diversity statistics, population growth indicators (Fu's F, Tajima's D) and genetic difference (F_st_) were calculated using ARLEQUIN 3.1 [36], assuming two populations of tigers (Indian subspecies and all other subspecies). To avoid the effects of related individuals in our analyses, we used STR data from 39 representative samples from all the areas sampled. For intra-population diversity statistics, samples were divided into Northern India (n = 10), Central India (n = 11) and Southern India (n = 18). Tests for selective neutrality were performed using DNASP 4.0 [37]. A statistical parsimony network based on 1263 bp mtDNA sequences of all the tiger subspecies was created using NETWORK (Fluxus Technology Ltd.). Two-population models were explored to estimate migration rate with the mitochondrial DNA and STR data with coalescent analysis program LAMARC and were based on genetic data from five common microsatellite loci.

#### Re-sampling to investigate the lower sample size outside India

If the higher number of haplotypes observed in Indian samples was due to a higher sample size, sampling fewer samples from the Indian haplotype distribution should result in fewer haplotypes. We used the observed haplotypic distribution from Indian tigers, and sampled (with replacement) 57 (total number of samples outside India) individuals for which we tabulated the number of haplotypes. This process is repeated 10,000 times. We then compared the number of haplotypes sampled in the simulation to the observed number of non-Indian haplotypes.

To determine the presence of hidden population structure within India, we performed a Bayesian clustering approach, as implemented in program STRUCTURE. We performed the analysis for K values between one and ten, using 50,000 iterations and a burn-in of 10,000 assuming correlated allele frequencies. The optimal value of K was selected based on Evanno *et al.* [38]. Ten repetitions for each value of K demonstrated similar results.

#### Analysis of past demography

We used five different approaches to detect past population demography. The first two approaches use summary statistics to detect population size changes, whereas the other three are likelihood or Bayesian methods. The summary statistic-based methods used were the Ewens, Watterson, Cornuet and Luikart method implemented in program BOTTLENECK [18], and the Garza-Williamson index or M ratio implemented in program ARLEQUIN [37]. The likelihood-based method used was the population growth rate estimation with program LAMARC [17]. The likelihood-based Bayesian methods used were implemented in the MSVAR programs [19],[20].

#### The Ewens, Watterson, Cornuet and Luikart approach [18]

This method uses two summary statistics of the allele frequency spectrum, number of alleles (n_A_) and expected heterozygosity (H_e_) to achieve the patterns expected for a demographically stable population. Simulations were performed under three mutation models: infinite allele model (IAM), single stepwise model (SMM) and two-phase model (TPM) to obtain the distribution of H_e_ and the values are then compared to the real data values. For TPM model, 30% multi-step mutation events were allowed during the simulations. This method can detect departures from mutation-drift equilibrium and neutrality, which can be explained by any departure from the null model, including selection, population growth or decline. More importantly, consistent results from independent loci could be attributed to demographic events over selection. Thus, this approach allows the detection of population size changes across different mutational models.

#### The Garza-Williamson index/M ratio approach [39]

This method uses data on the frequency and the total number of alleles and the allelic size range to investigate population decline. In a reducing population, the expectation of the reduction of number of alleles is much higher than the reduction of allelic size range. Thus the ratio between the number of alleles and the allelic size range is expected to be smaller in recently reduced populations than in equilibrium populations.

#### Likelihood approach (LAMARC) [17]

This method provides maximum likelihood estimates of growth (or decline) rates using a Metropolis-Hastings Monte Carlo Markov Chain algorithm. As it is a time backward approach, a negative value of g indicates that the population has been shrinking (it was bigger in the past than it is now) and a positive value indicates that it has been growing (it was smaller in the past than it is now). Genetic data from ten microsatellite loci was used for these analyses.

#### The Beaumont approach [19]

This approach assumes that a stable population of size N_1_ started to change (either decrease or increase) t_a_ generations ago to the current population size N_0_. This change in the population size is assumed to be either linear or exponential under stepwise mutation model (SMM). This bayesian approach uses the information from the full allelic distribution in a coalescent framework to estimate the posterior probability distribution of (i) r = N_0_/N_1_ (rate of population size change), (ii) t_f_ = t_a_/N_0_ (time since the population size change started, scaled by N_0_), and (iii) θ = 2N_0_μ. A Markov Chain Monte Carlo (MCMC) algorithm is used to generate samples from the posterior distribution of these parameters. Although this method allows the quantification of a population increase or decrease, N_0_ and N_1_ cannot be estimated independently. Similarly, it can only approximate t_a_ as a time scaled by N_0_, with N_0_ being unknown. Thus, the population size change can be quantified, but it cannot be dated. This model was employed to test for a genetic bottleneck under different model of population size change (linear or exponential).

For each analysis, at least three independent runs were performed using different parameter configurations and starting values. Most importantly, all the runs were carried out with positive starting values of log(r) (see Table S4 and 5), corresponding to a population expansion of various levels. This avoids favouring regions of the parameter space corresponding to a population decline.

Rectangular prior distributions were assumed for log(r), log(θ) and log(t_f_). Wide bounds were chosen (between 10^−3^ and 10^3^ on a natural log scale) for minimum effects on posterior distributions.

#### The Storz and Beaumont approach [20]

This approach is an extension of Beaumont's method and allows quantification of effective population sizes N_0_ and N_1_, rather than their ratio along with T, time since the population change. The method assumes an exponential model of population size change. In this model, prior distributions for N_0_, N_1_, T and μ are assumed to be log normal. The mean and the standard deviations of these prior log normal distributions are drawn from priors (or hyperpriors) distributions.

Wide “uninformative” priors were used to perform multiple runs for this approach (Table S6). For minimal effect towards the posterior distributions variances for the prior distributions were kept large. The total number of iterations was always 2 × 10^6^.

The generation time for tigers is known to be about five years [25], and we used this information for all analyses.

## Supporting Information

Figure S1 Figure showing investigated mitochondrial regions and the variable positions (lines) and the associated heterozygosities of these positions for all tiger subspecies. The colored boxes highlight the regions amplified by our primers for this study.
(0.52 MB TIF)

Figure S2 (A) Population size change for the tigers in the Indian subcontinent, with black and green curves corresponding to the posterior distributions under models of exponential and linear population size change, respectively. The prior distribution is represented by flat dotted line. Irrespective of various models, there is no support for population increase. (B) The posterior distributions for ancestral (red curve) and present (green) population size are represented here. The priors are represented by the dotted line (present population) and dashed line (ancestral population). (C) The posterior distribution for the time since the population decline started for Indian tigers (black curve) is represented here. The priors are shown by the dashed lines. The distribution has a median value at around 270 years. (D) Joint posterior distribution of ancestral and current population size based on South and Central India tiger data. The 90%, 50%, and 10% highest probability density (HPD) limits are plotted for the joint distribution of ancestral and current population size on a logarithmic scale. The diagonal line corresponds to stable population size.
(1.34 MB TIF)

Figure S3 (A) Population size change for the Indian tigers (30 microsatellites, n = 5) with red and green curves corresponding to the posterior distributions under models of exponential and linear population size change, respectively. The prior distribution is represented by flat dotted line. (B) The posterior distributions for ancestral (red curve) and present (green) population size are represented here (30 microsatellites, n = 5). The priors are represented by the dotted line (present population) and dashed line (ancestral population). (C) The posterior distribution for the time since the population decline started for Indian tigers (black curve) is represented here. The priors are shown by the dashed lines. The distribution has a median value at around 258 years. (D) Joint posterior distribution of ancestral and current population size based on Indian tiger data. The 90%, 50%, and 10% highest probability density (HPD) limits are plotted for the joint distribution of ancestral and current population size on a logarithmic scale. The diagonal line corresponds to stable population size.
(1.28 MB TIF)

Figure S4 (A) Posterior distributions for rate of population size changes from independent MCMC runs for Indian tigers under linear change model (Beaumont method). Details of the models are given in Table S4. (B) Posterior distributions for rate of population size changes from independent MCMC runs for Indian tigers under exponential change model (Beaumont method). Details of the models are given in Table S5.
(0.68 MB TIF)

Figure S5 (A) Posterior distributions for current population size from independent MCMC runs for Indian tigers under Storz and Beaumont method. Details of the models are given in Table S6. (B) Posterior distributions for ancestral population size from independent MCMC runs for Indian tigers under Storz and Beaumont method. Details of the models are given in Table S6. (C) Posterior distributions for time since population decline from independent MCMC runs for Indian tigers under Storz and Beaumont method. Details of the models are given in Table S6.
(0.64 MB TIF)

Figure S6 (A) Population size change for the Indo-Chinese tigers (*P. t. corbetti*) (30 microsatellites, n = 27) with red and green curves corresponding to the posterior distributions under models of exponential and linear population size change, respectively. The prior distribution is represented by flat dotted line. (B) The posterior distributions for ancestral (red curve) and present (green) population size are represented here (30 microsatellites, n = 27). The priors are represented by the dotted line (present population) and dashed line (ancestral population). (C) The posterior distribution for the time since the population decline started for Indo-Chinese tigers (black curve) is represented here. The priors are shown by the dashed lines. The distribution has a median value at around 158 years. (D) Joint posterior distribution of ancestral and current population size based on Indian tiger data. The 90%, 50%, and 10% highest probability density (HPD) limits are plotted for the joint distribution of ancestral and current population size on a logarithmic scale. The diagonal line corresponds to stable population size.
(1.29 MB TIF)

Figure S7 (A) Posterior distributions for rate of population size changes from independent MCMC runs for Indo-Chinese tigers under linear change model (Beaumont method). Details of the models are given in Table S4. (B) Posterior distributions for rate of population size changes from independent MCMC runs for Indo-Chinese tigers under exponential change model (Beaumont method). Details of the models are given in Table S5.
(0.66 MB TIF)

Figure S8 (A) Posterior distributions for current population size from independent MCMC runs for Indo-Chinese tigers under Storz and Beaumont method. Details of the models are given in Table S6. (B) Posterior distributions for ancestral population size from independent MCMC runs for Indo-Chinese tigers under Storz and Beaumont method. Details of the models are given in Table S6. (C) Posterior distributions for time since population decline from independent MCMC runs for Indo-Chinese tigers under Storz and Beaumont method. Details of the models are given in Table S6.
(0.64 MB TIF)

Table S1 Information on samples used in this study.
(0.11 MB DOC)

Table S2 Species-specific mitochondrial primers designed and used in this study.
(0.05 MB DOC)

Table S3 Information of 10 microsatellite loci used in this study.
(0.05 MB DOC)

Table S4 Linear models (Beaumont method).
(0.04 MB DOC)

Table S5 Exponential models (Beaumont method).
(0.04 MB DOC)

Table S6 Exponential models (Storz and Beaumont method).
(0.05 MB DOC)
