# Correction: Why the Indian Subcontinent Holds the Key to Global Tiger Recovery

**DOI:** 10.1371/annotation/9f8748f6-300f-450e-bbed-63e66e1b6661

**Published:** 2009-08-24

**Authors:** Samrat Mondol, K. Ullas Karanth, Uma Ramakrishnan

The following information was missing from the Funding section: SM was supported by a Department of Science and Technology grant to UR. Field sampling was partially funded by WCS.

