# Supplementary figures and images for: Why the Indian Subcontinent Holds the Key to Global Tiger Recovery
Source: PLoS Genet. 2009 Aug 14;5(8):e1000585. doi: 10.1371/journal.pgen.1000585 (PMC2716534; doi:10.1371/journal.pgen.1000585)

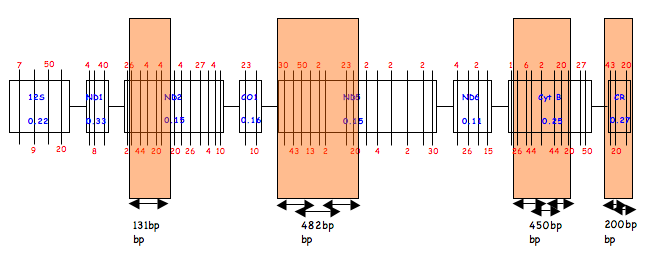

Supplement: Figure S1 — Figure showing investigated mitochondrial regions and the variable positions (lines) and the associated heterozygosities of these positions for all tiger subspecies. The colored boxes highlight the regions amplified by our primers for this study. (0.52 MB TIF) [file pgen.1000585.s001.tif]

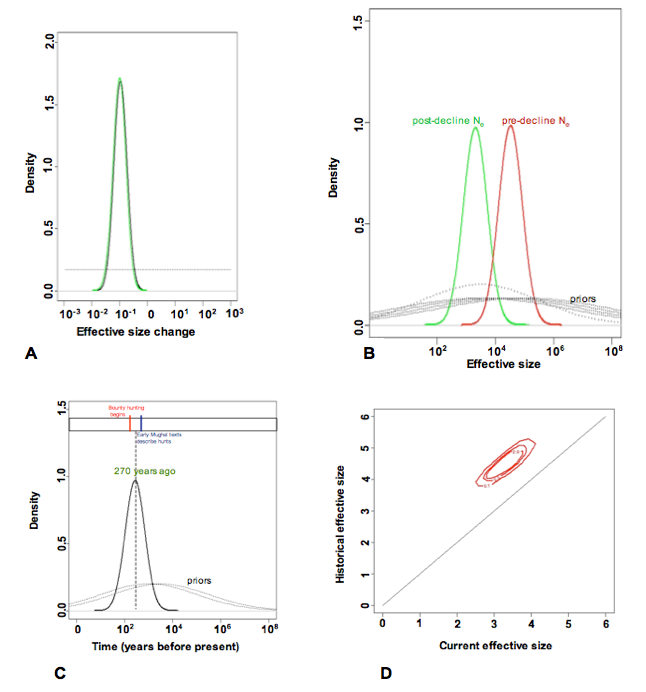

Supplement: Figure S2 — (A) Population size change for the tigers in the Indian subcontinent, with black and green curves corresponding to the posterior distributions under models of exponential and linear population size change, respectively. The prior distribution is represented by flat dotted line. Irrespective of various models, there is no support for population increase. (B) The posterior distributions for ancestral (red curve) and present (green) population size are represented here. The priors are represented by the dotted line (present population) and dashed line (ancestral population). (C) The posterior distribution for the time since the population decline started for Indian tigers (black curve) is represented here. The priors are shown by the dashed lines. The distribution has a median value at around 270 years. (D) Joint posterior distribution of ancestral and current population size based on South and Central India tiger data. The 90%, 50%, and 10% highest probability density (HPD) limits are plotted for the joint distribution of ancestral and current population size on a logarithmic scale. The diagonal line corresponds to stable population size. (1.34 MB TIF) [file pgen.1000585.s002.tif]

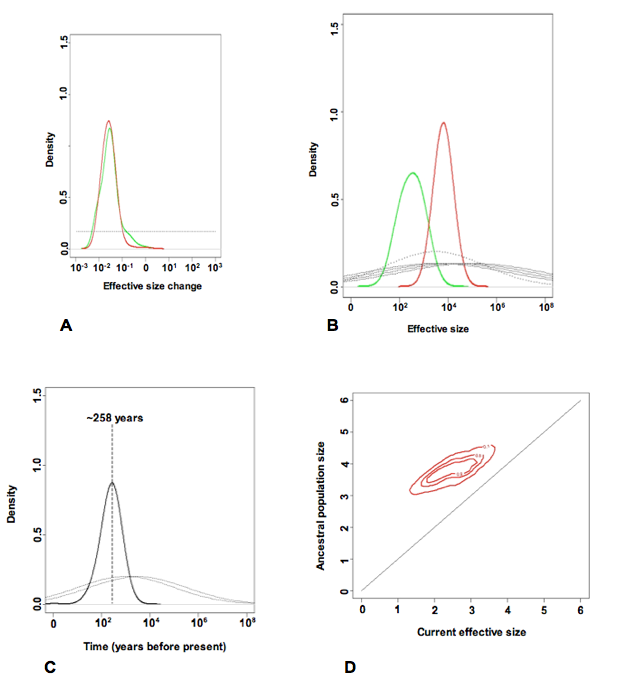

Supplement: Figure S3 — (A) Population size change for the Indian tigers (30 microsatellites, n = 5) with red and green curves corresponding to the posterior distributions under models of exponential and linear population size change, respectively. The prior distribution is represented by flat dotted line. (B) The posterior distributions for ancestral (red curve) and present (green) population size are represented here (30 microsatellites, n = 5). The priors are represented by the dotted line (present population) and dashed line (ancestral population). (C) The posterior distribution for the time since the population decline started for Indian tigers (black curve) is represented here. The priors are shown by the dashed lines. The distribution has a median value at around 258 years. (D) Joint posterior distribution of ancestral and current population size based on Indian tiger data. The 90%, 50%, and 10% highest probability density (HPD) limits are plotted for the joint distribution of ancestral and current population size on a logarithmic scale. The diagonal line corresponds to stable population size. (1.28 MB TIF) [file pgen.1000585.s003.tif]

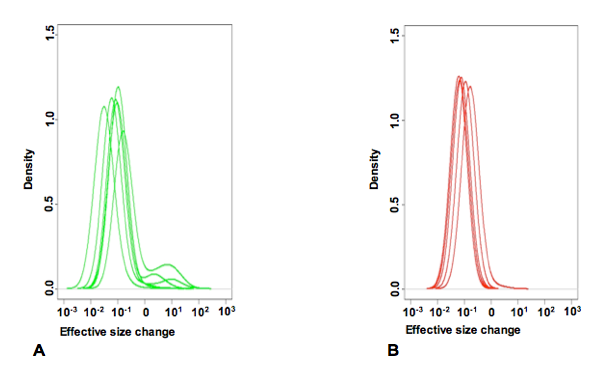

Supplement: Figure S4 — (A) Posterior distributions for rate of population size changes from independent MCMC runs for Indian tigers under linear change model (Beaumont method). Details of the models are given in Table S4. (B) Posterior distributions for rate of population size changes from independent MCMC runs for Indian tigers under exponential change model (Beaumont method). Details of the models are given in Table S5. (0.68 MB TIF) [file pgen.1000585.s004.tif]

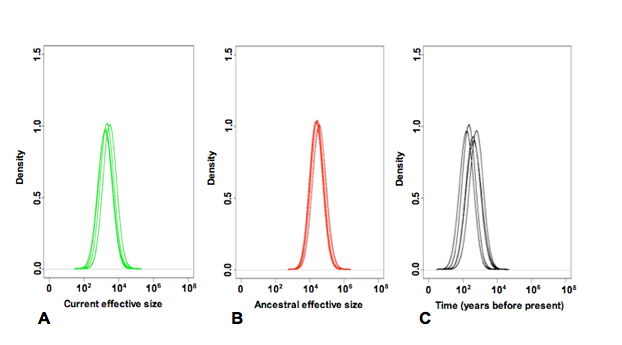

Supplement: Figure S5 — (A) Posterior distributions for current population size from independent MCMC runs for Indian tigers under Storz and Beaumont method. Details of the models are given in Table S6. (B) Posterior distributions for ancestral population size from independent MCMC runs for Indian tigers under Storz and Beaumont method. Details of the models are given in Table S6. (C) Posterior distributions for time since population decline from independent MCMC runs for Indian tigers under Storz and Beaumont method. Details of the models are given in Table S6. (0.64 MB TIF) [file pgen.1000585.s005.tif]

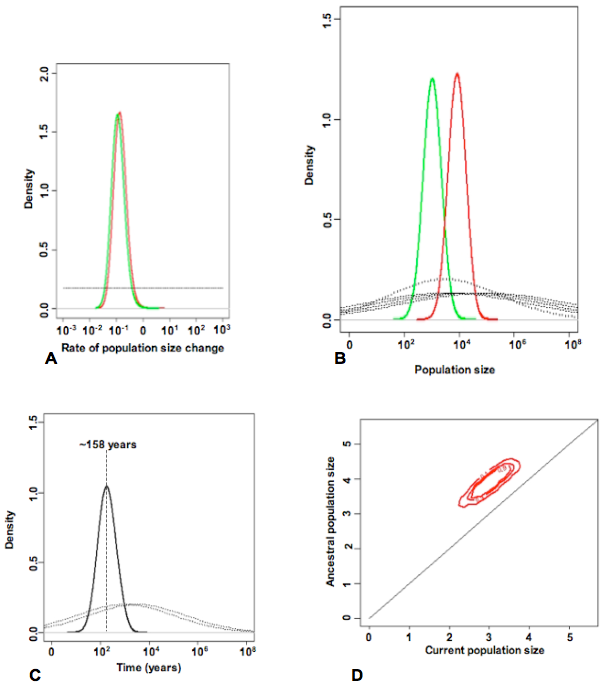

Supplement: Figure S6 — (A) Population size change for the Indo-Chinese tigers (P. t. corbetti) (30 microsatellites, n = 27) with red and green curves corresponding to the posterior distributions under models of exponential and linear population size change, respectively. The prior distribution is represented by flat dotted line. (B) The posterior distributions for ancestral (red curve) and present (green) population size are represented here (30 microsatellites, n = 27). The priors are represented by the dotted line (present population) and dashed line (ancestral population). (C) The posterior distribution for the time since the population decline started for Indo-Chinese tigers (black curve) is represented here. The priors are shown by the dashed lines. The distribution has a median value at around 158 years. (D) Joint posterior distribution of ancestral and current population size based on Indian tiger data. The 90%, 50%, and 10% highest probability density (HPD) limits are plotted for the joint distribution of ancestral and current population size on a logarithmic scale. The diagonal line corresponds to stable population size. (1.29 MB TIF) [file pgen.1000585.s006.tif]

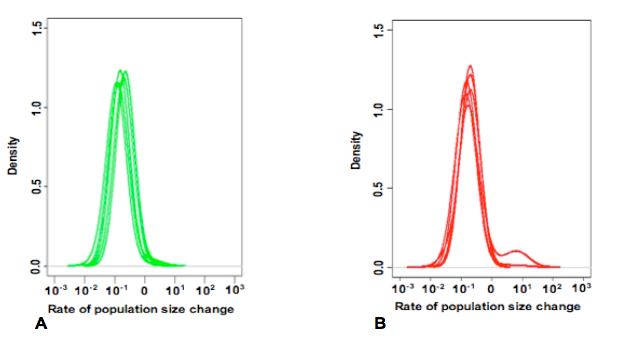

Supplement: Figure S7 — (A) Posterior distributions for rate of population size changes from independent MCMC runs for Indo-Chinese tigers under linear change model (Beaumont method). Details of the models are given in Table S4. (B) Posterior distributions for rate of population size changes from independent MCMC runs for Indo-Chinese tigers under exponential change model (Beaumont method). Details of the models are given in Table S5. (0.66 MB TIF) [file pgen.1000585.s007.tif]

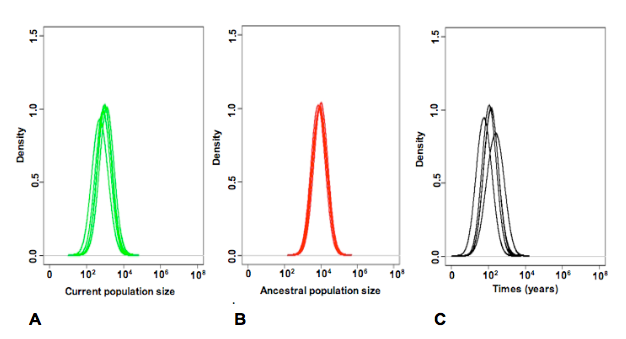

Supplement: Figure S8 — (A) Posterior distributions for current population size from independent MCMC runs for Indo-Chinese tigers under Storz and Beaumont method. Details of the models are given in Table S6. (B) Posterior distributions for ancestral population size from independent MCMC runs for Indo-Chinese tigers under Storz and Beaumont method. Details of the models are given in Table S6. (C) Posterior distributions for time since population decline from independent MCMC runs for Indo-Chinese tigers under Storz and Beaumont method. Details of the models are given in Table S6. (0.64 MB TIF) [file pgen.1000585.s008.tif]
