# Supplementary material for: Why the Indian Subcontinent Holds the Key to Global Tiger Recovery
Source: PLoS Genet. 2009 Aug 14;5(8):e1000585. doi: 10.1371/journal.pgen.1000585 (PMC2716534; doi:10.1371/journal.pgen.1000585)
Supplement: Table S1 — Information on samples used in this study. (0.11 MB DOC) [file pgen.1000585.s009.doc]

| Haplotype  Table S1: Information on samples used in this study | Locale of Origin | Genbank accession no. |
| --- | --- | --- |
| TIG21* ^1^ | Kalakkad Mundanthurai tiger reserve, Kerala | EU661619, EU661640, EU661661, EU661682 |
| TIG15* ^2^ | Kalakkad Mundanthurai tiger reserve, Kerala | EU661613, EU661634, EU661655, EU661676 |
| TIG15^2^ | Periyar national park, Kerala |  |
| TIG14* ^2^ | Parambikkulam wildlife sanctuary, Kerala | EU661612, EU661633, EU661654, EU661675 |
| TIG11^1^ | Mudumalai wildlife sanctuary, Tamil Nadu |  |
| TIG16* ^1^ | Bandipur national park, Karnataka | EU661614, EU661635, EU661656, EU661677 |
| TIG19* ^1^ | Bandipur national park, Karnataka | EU661617, EU661638, EU661659, EU661680 |
| TIG13* ^2^ | Bandipur national park, Karnataka | EU661611, EU661632, EU661653, EU661674 |
| TIG11^2^ | Biligiri Rangaswamy Temple Wildlife Sanctuary, Karnataka |  |
| TIG11^1^ | Nagarhole national park, Karnataka |  |
| TIG28* ^2^ | Nagarhole national park, Karnataka | EU661626, EU661647, EU661668, EU661688 |
| TIG11^2^ | Nagarhole national park, Karnataka |  |
| TIG11^2^ | Brahmagiri Wildlife Sanctuary, Karnataka |  |
| TIG11^2^ | Brahmagiri Wildlife Sanctuary, Karnataka |  |
| TIG18* ^1^ | Bhadra wildlife sanctuary, Karnataka | EU661616, EU661637, EU661658, EU661679 |
| TIG18^1^ | Bhadra wildlife sanctuary, Karnataka |  |
| TIG11^2^ | Bhadra wildlife sanctuary, Karnataka |  |
| TIG11^2^ | Kudremukh national park, Karnataka |  |
| TKDRM2^1^ | Kudremukh national park, Karnataka |  |
| TIG11^2^ | Kushalnagar, Karnataka |  |
| CWST1^3^ | Doddaballapur, Karnataka (Tissue) |  |
| TIG22* ^2^ | Dandeli wildlife sanctuary, Karnataka | EU661620, EU661641, EU661662, FJ228452 |
| TIG11^2^ | Nilgiri wildlife sanctuary, Tamil Nadu |  |
| TIG22^1^ | Belgaum, Karnataka |  |
| TIG30* ^2^ | Raipur Zoo, Chattishgarh (wild caught) | EU661628, EU661649, EU661670, EU661690 |
| TIG20* ^2^ | Tadoba national park, Maharastra | EU661618, EU661639, EU661660, EU661681 |
| TIG17* ^2^ | Tadoba national park, Maharastra (Tissue) | EU661615, EU661636, EU661657, EU661678 |
| TIG12* ^1^ | Tadoba national park, Maharastra | EU661610, EU661631, EU661652, EU661673 |
| TIG11^1^ | Pench national park, Maharastra |  |
| TIG6^§2^ | Melghat tiger reserve, Maharastra |  |
| TIG27* ^1^ | Melghat tiger reserve, Maharastra | EU661625, EU661646, EU661667, EU661687 |
| TIG31* ^1^ | Kanha national park, Madhya Pradesh | EU661629, EU661650, EU661671, EU661691 |
| TIG32* ^2^ | Kanha national park, Madhya Pradesh | Genbank accession number awaiting |
| TIG33* ^2^ | Kanha national park, Madhya Pradesh | Genbank accession number awaiting |
| TIG32 ^1^ | Kanha national park, Madhya Pradesh |  |
| TKNHR2^3^ | Kanha national park, Madhya Pradesh |  |
| TKNHR6^3^ | Kanha national park, Madhya Pradesh |  |
| TKHNR7^3^ | Kanha national park, Madhya Pradesh |  |
| TIG11^2^ | Bandhavgarh national park. Madhya Pradesh |  |
| TIG11^2^ | Nagarjunsagar-Srisailam tiger reserve,  Andhra Pradesh |  |
| TIG11^1^ | Nagarjunsagar-Srisailam tiger reserve,  Andhra Pradesh |  |
| TIG11* ^2^ | Nagarjunsagar-Srisailam tiger reserve,  Andhra Pradesh | EU661609,EU661630,EU661651,EU661672 |
| TIG34*^1^ | Nagarjunsagar-Srisailam tiger reserve,  Andhra Pradesh | Genbank accession number awaiting |
| TIG35*^1^ | Nagarjunsagar-Srisailam tiger reserve,  Andhra Pradesh | Genbank accession number awaiting |
| TIG36*^1^ | Nagarjunsagar-Srisailam tiger reserve,  Andhra Pradesh | Genbank accession number awaiting |
| TNSTR7^3^ | Nagarjunsagar-Srisailam tiger reserve,  Andhra Pradesh |  |
| TIG23^2^ | Ranthambhore national park, Rajasthan |  |
| TIG30^2^ | Ranthambhore national park, Rajasthan |  |
| TIG30^2^ | Ranthambhore national park, Rajasthan |  |
| TIG23* ^1^ | Sunderbans national park, West Bengal | EU661621, EU661642, EU661663, EU661683 |
| TIG29* ^2^ | Sunderbans national park, West Bengal | EU661627, EU661648, EU661669, EU661689 |
| TIG24* ^1^ | Kaziranga national park, Assam | EU661622, EU661643, EU661664, EU661684 |
| TIG25* ^2^ | Manas national park, Assam | EU661623, EU661644, EU661665, EU661685 |
| TIG37* ^2^ | Manas national park, Assam | Genbank accession number awaiting |
| TIG38* ^2^ | Manas national park, Assam | Genbank accession number awaiting |
| TIG25^2^ | Orang national park, Assam |  |
| TIG39* ^2^ | HuKuang tiger reserve, Myanmar | Genbank accession number awaiting |
| TIG38 ^1^ | HuKuang tiger reserve, Myanmar |  |
| TIG38 ^2^ | HuKuang tiger reserve, Myanmar |  |
| TIG26* ^2^ | Satkosia gorge sanctuary, Orissa | EU661624, EU661645, EU661666, EU661686 |
| A1^3^ | Bandipura national park, Karnataka |  |
| A3^3^ | Bandipura national park, Karnataka |  |
| A4^3^ | Bandipura national park, Karnataka |  |
| A9^3^ | Bandipura national park, Karnataka |  |
| A12^3^ | Bandipura national park, Karnataka |  |
| A15^3^ | Bandipura national park, Karnataka |  |
| A14(N)^3^ | Bandipura national park, Karnataka |  |
| A15(N) ^3^ | Bandipura national park, Karnataka |  |
| A17(N) ^3^ | Bandipura national park, Karnataka |  |
| NG3^3^ | Nagarhole national park, Karnataka |  |
| NG10^3^ | Nagarhole national park, Karnataka |  |
| NG11^3^ | Nagarhole national park, Karnataka |  |
| NG13^3^ | Nagarhole national park, Karnataka |  |

^1^ - Samples with only mitochondrial data

^2^ - Samples with both mitochondrial and microsatellite data

^3^ - Samples with only microsatellite data

*- Sequences of particular haplotypes submitted to Genbank. Other samples with same haplotypes are mentioned

§- Sequence matched with haplotype TIG6 from NCBI (Luo *et al.*)

Genetic data from wild caught tigers from all the subspecies was used from Luo *et al.*[13]. Genotype data was kindly provided by Shu-Jin Luo*.* The samples we incorporated into our analysis were as follows:

*P. t. altaica*: Pti-111, Pti-112, Pti-113, Pti-114, Pti-115, Pti-117, Pti-118, Pti-120, Pti-122, Pti-123, Pti-124, Pti-126, Pti-127, Pti-128, Pti-131, Pti-133, Pti-134, Pti-135, Pti-154.

*P. t. corbetti*: Pti-249, Pti-250, Pti-290, Pti-291, Pti-292, Pti-296, Pti-306, Pti-307, Pti-315, Pti-316, Pti-CB11, Pti-CB13, Pti-CB14, Pti-CB15, Pti-CB16, Pti-CB18, Pti-CB22, Pti-CB24, Pti-CB32, Pti-CB36, Pti-CB6, Pti-CB7, Pti-CB23, Pti-CB27, Pti-CB29, Pti-CB31, Pti-CB34.

*P. t. jacksoni*: Pti-210, Pti-304, Pti-267, Pti-263, Pti-163, Pti-265, Pti-266, Pti-262, Pti-264, Pti-268, Pti-271.

*P. t. sumatrae*: Pti-170, Pti-171, Pti-172, Pti-174, Pti-178, Pti-181, Pti-183, Pti-184, Pti-185, Pti-186, Pti-216.

*P. t. tigris*: Pti-102, Pti-103, Pti-104, Pti-105, Pti-165.
