# Supplementary material for: Why the Indian Subcontinent Holds the Key to Global Tiger Recovery
Source: PLoS Genet. 2009 Aug 14;5(8):e1000585. doi: 10.1371/journal.pgen.1000585 (PMC2716534; doi:10.1371/journal.pgen.1000585)
Supplement: Table S2 — Species-specific mitochondrial primers designed and used in this study. (0.05 MB DOC) [file pgen.1000585.s010.doc]

Table S2- Species-specific mitochondrial primers designed and used in this study

| Primer  Name | Primer sequence | Amplicon size  (bp) |
| --- | --- | --- |
| TIGND2 F1 | TAGTCTGAATCGGCTTCG | 195 |
| TIGND2 R1 | CCGTTATAATGGATGCCA |  |
| TIGND5 F1 | GCCCCTATATTAACCAGT | 195 |
| TIGND5 R1 | ATCCTACATCTCCAATAC |  |
| TIGND5 F2 | TATCAGACGCAAACACTG | 224 |
| TIGND5 R2 | AATAAAGCGGAGACGGGA |  |
| TIGND5 F3 | ACCTACACCCATGATTGC | 187 |
| TIGND5 R3 | TTTTGTGTGAGGGCACAG |  |
| TIGCYT B F2 | CGTCTGTCTATACATGCA | 200 |
| TIGCYT B R2 | TACTCTACTAGGTCGGTC |  |
| TIGCYT B F3 | ATGTCTTTTTGAGGGGCA | 191 |
| TIGCYT B R3 | GTATTGGATCCTGTTTCG |  |
| TIGCYT B F4 | TTAACCCTAGCAGCAGTC | 184 |
| TIGCYT B R4 | TGTAGTTATCAGGGTCTC |  |
| TIGCR F1 | GGGAAGGAGAATATGTAC | 142 |
| TIGCR R1 | CACAGAACGGGTATATGC |  |
| TIGCR F2 | CGAAAACAACCCCATGAC | 137 |
| TIGCR R2 | GCTTCGTGTTGTGTGTTC |  |
