# Supplementary material for: Why the Indian Subcontinent Holds the Key to Global Tiger Recovery
Source: PLoS Genet. 2009 Aug 14;5(8):e1000585. doi: 10.1371/journal.pgen.1000585 (PMC2716534; doi:10.1371/journal.pgen.1000585)
Supplement: Table S3 — Information of 10 microsatellite loci used in this study. (0.05 MB DOC) [file pgen.1000585.s011.doc]

Table S3- Information of 10 microsatellite loci used in this study

| Locus | Product size range  (bp) | Amplification success  (%) | Allelic dropout (%) | False alleles | No. of alleles |
| --- | --- | --- | --- | --- | --- |
| FCA126* | 112-156 | 100 | 0 | 0 | 11 |
| FCA069* | 85-107 | 100 | 0 | 0 | 11 |
| FCA090* | 91-117 | 100 | 0 | 0 | 13 |
| FCA304* | 105-139 | 98 | 0 | 0 | 19 |
| FCA441* | 138-166 | 98 | 0.5 | 0 | 8 |
| FCA672 | 82-114 | 98 | 0 | 0 | 14 |
| FCA628 | 84-114 | 100 | 0 | 0 | 15 |
| FCA232 | 86-118 | 98 | 0 | 0 | 13 |
| FCA230 | 93-129 | 95 | 0 | 0 | 11 |
| FCA279 | 89-107 | 93 | 1.2 | 0 | 10 |

- Loci comparable with the outside tigers
