# Supplementary material for: Why the Indian Subcontinent Holds the Key to Global Tiger Recovery
Source: PLoS Genet. 2009 Aug 14;5(8):e1000585. doi: 10.1371/journal.pgen.1000585 (PMC2716534; doi:10.1371/journal.pgen.1000585)
Supplement: Table S4 — Linear models (Beaumont method). (0.04 MB DOC) [file pgen.1000585.s012.doc]

Table S4 – Linear models (Beaumont method)

| Linear models | log(*θ*) | log(*r*) | log(*t_f_*) | Range | thinning | steps | iterations |
| --- | --- | --- | --- | --- | --- | --- | --- |
| Run_01 | 10 | 10 | 100 | 10^-3^- 10^-3^ | 10^3^ | 2 10^3^ | 2 10^6^ |
| Run_02 | 100 | 10 | 100 | 10^-3^- 10^-3^ | 10^3^ | 2 10^3^ | 2 10^6^ |
| Run_03 | 10 | 100 | 10 | 10^-3^- 10^-3^ | 10^3^ | 2 10^3^ | 2 10^6^ |
| Run_04 | 10 | 100 | 1000 | 10^-3^- 10^-3^ | 10^3^ | 2 10^3^ | 2 10^6^ |
| Run_05 | 100 | 100 | 1000 | 10^-3^- 10^-3^ | 10^3^ | 2 10^3^ | 2 10^6^ |
| Run_06 | 10 | 1000 | 10000 | 10^-3^- 10^-3^ | 10^3^ | 2 10^3^ | 2 10^6^ |
| Run_07 | 10 | 10000 | 1000 | 10^-3^- 10^-3^ | 10^3^ | 2 10^3^ | 2 10^6^ |
