# Supplementary material for: Why the Indian Subcontinent Holds the Key to Global Tiger Recovery
Source: PLoS Genet. 2009 Aug 14;5(8):e1000585. doi: 10.1371/journal.pgen.1000585 (PMC2716534; doi:10.1371/journal.pgen.1000585)
Supplement: Table S6 — Exponential models (Storz and Beaumont method). (0.05 MB DOC) [file pgen.1000585.s014.doc]

Table S6 – Exponential models (Storz and Beaumont method)

| Runs | log(*N_0_*) | log(*N_1_*) | log(*θ*) | log(*T*) | log(*N_0_*) | log(*N_1_*) | log(*θ*) | log(*T*) |
| --- | --- | --- | --- | --- | --- | --- | --- | --- |
| Run 01 | 4 1 | 4 1 | -3.5 1 | 3 1 | 3.5 2 0 0.5 | 5 3 0 0.5 | -3.5 0.25 0 0.5 | 3 2 0 0.5 |
| Run 02 | 4 1 | 4 1 | -3.5 1 | 3 1 | 3.5 2 0 0.5 | 4.5 3 0 0.5 | -3.5 0.25 0 0.5 | 3 2 0 0.5 |
| Run 03 | 4 1 | 4 1 | -3.5 1 | 3 1 | 3.5 2 0 0.5 | 4 3 0 0.5 | -3.5 0.25 0 0.5 | 3 2 0 0.5 |
| Run 04 | 4 1 | 4 1 | -3.5 1 | 3 1 | 3.5 2 0 0.5 | 3.5 3 0 0.5 | -3.5 0.25 0 0.5 | 3.5 2 0 0.5 |
| Run 05 | 4 1 | 4 1 | -3.5 1 | 2 1 | 3.5 2 0 0.5 | 3.5 3 0 0.5 | -3.5 0.25 0 0.5 | 3 2 0 0.5 |
| Run 06 | 4 1 | 4 1 | -3.5 1 | 3 1 | 3.5 2 0 0.5 | 3.5 3 0 0.5 | -3.5 0.25 0 0.5 | 3 2 0 0.5 |
